# The ecology of polyploid establishment and exclusion, with implications for polyploid biogeography

**DOI:** 10.1111/nph.20451

**Published:** 2025-02-10

**Authors:** Wilhelm H. A. Osterman, James G. Hagan, Jeannette Whitton, Anne D. Bjorkman

**Affiliations:** ^1^ Department of Biological and Environmental Sciences University of Gothenburg Gothenburg 413 90 Sweden; ^2^ Gothenburg Global Biodiversity Centre Gothenburg 413 90 Sweden; ^3^ Department of Marine Sciences University of Gothenburg Gothenburg 413 90 Sweden; ^4^ Department of Botany and Biodiversity Research Centre The University of British Columbia Vancouver V6T 1Z4 Canada

**Keywords:** whole‐genome duplication, vegetative reproduction, pollination, clonality, triploidy, macroecology, abiotic stress, arctic polyploids

## Abstract

The relationship between polyploid formation, triploid fitness and plant reproduction has been studied for over a century, and uniparental reproduction has long been recognized to play a crucial role in polyploid establishment. Yet, we lack a synthesized framework of how polyploid establishment is expected to be influenced by different reproductive modes among angiosperms. Here, we provide new perspectives on how uniparental reproduction, pollination ecology, triploid fitness and assortative mating can impact minority cytotype exclusion (MCE) and, thereby, the likelihood of polyploid establishment. We review the current state of knowledge of the reproductive mechanisms that impact polyploid establishment and discuss often overlooked aspects of these processes, such as the influence of pollinator communities on rates of self‐pollination. We propose a framework for considering how variation in reproductive strategies and pollinator communities can impact the ability of a polyploid to overcome MCE. Finally, we propose links between patterns of variation in uniparental reproduction across plant communities and observed patterns in the distribution and abundance of polyploids.

## Introduction

### Background

Whole‐genome duplication is a major force impacting the ecology and evolution of angiosperms, with estimates that up to one‐third of all angiosperms are recent polyploids (Wood *et al*., 2009; Heslop‐Harrison *et al*., 2023). Decades of research have aimed to advance our understanding of polyploid evolution, addressing how and when polyploids establish, and exploring global patterns of polyploid distribution. Despite these efforts, the reasons for the increase in polyploid frequency at higher latitudes remain unclear (Rice *et al*., 2019). It is commonly suggested that polyploids survive better under stressful environmental conditions than their nonpolyploid progenitors (Bomblies, 2020; Van de Peer *et al*., 2021), and that this could underlie the observed latitudinal gradient in polyploid frequency. Alternatively, the uneven distribution of polyploids across the globe could be caused by variation in the frequency of polyploid formation (Gustafsson, 1948), including stochastic effects (Gerstner *et al*., 2024), but the connections among these explanations remain poorly understood. Given the abundance of polyploid angiosperms, our understanding of global patterns of plant diversity will benefit from understanding the biological processes that promote or limit polyploid success (Box 1).

Box 1 **Glossary**

**Allopolyploidy:** polyploidy that arises following hybridization (in contrast to autopolyploidy).

**Assortative mating (=positive assortative mating):** mating within a cytotype more frequently than expected by chance.

**Autonomous selfing:** self‐pollination that occurs in absence of a vector.

**Autopolyploidy:** polyploidy arising within a species (in contrast to allopolyploidy).

**Cytotype:** ploidy variant within a species or lineage.

**Dioecy:** the condition of having male and female flowers on separate plants.

**Geitonogamy:** self‐pollination between flowers on the same individual, including ramets of the same clone.

**Minority cytotype exclusion:** the loss of a cytotype driven by frequency‐dependent mating disadvantages.

**Postzygotic isolation:** reproductive isolation acting after zygote formation, including the production of inviable or sterile offspring from inter‐cytotype mating.

**Prezygotic isolation:** reproductive isolation that is expressed before zygote formation, lower fertilization success from inter‐cytotype pollination.

**Selfing/Self‐pollination:** uniparental sexual reproduction resulting from the fusion of pollen and ovules of the same individual.

**Unreduced gamete (UG):** pollen or ovules that have not undergone meiotic reduction (e.g. 2n pollen produced by a 2n plant).

One process widely recognized to impact the establishment and persistence of polyploid populations is the frequency‐dependent disadvantage called minority cytotype exclusion (MCE; Levin, 1975). Under MCE, the ability of individuals of the minority cytotype to produce viable and fertile sexual offspring is reduced because they mainly receive pollen from the common cytotype. If mating is random between common diploids and newly formed rare tetraploids, tetraploid individuals will experience high rates of hybridization, mostly yielding inviable or infertile triploids and leading to the exclusion of tetraploids from the population (Ramsey & Schemske, 1998; Köhler *et al*., 2010). Recently formed polyploids must therefore overcome the minority cytotype disadvantage to establish (Husband, 2000; Sutherland *et al*., 2020). Given the importance of MCE in forming polyploid populations, whether and how MCE might contribute to latitudinal patterns of polyploid diversity merits consideration.

### Overcoming MCE as a polyploid

The strong reproductive disadvantage caused by MCE has led many to argue that polyploid establishment must hinge on large fitness advantages (Burton & Husband, 2000; van de Peer *et al*., 2021). For example, polyploidization can produce neopolyploids that are more tolerant of environmental stress, such as drought or salinity (Bomblies, 2020; Van de Peer *et al*., 2021). However, synthetic neopolyploids generally have lower reproductive fitness than diploid progenitors (Clo & Kolář, 2021) and thus, whether increased tolerance would have large enough effects on fitness to compensate for the effects MCE is unclear. An alternative way to overcome MCE that does not rest only on fitness differences is for polyploids to reproduce more frequently via uniparental reproduction, increasing their probability of establishment through increased clonality or selfing (Gustafsson, 1948; Levin, 1975; Husband, 2000). Associations between uniparental reproduction and polyploidy have been reported repeatedly, including evidence that polyploids have increased levels of self‐pollination, higher prevalence of self‐compatibility, increased vegetative reproduction, and increased prevalence of apomixis (Barringer, 2007; Whitton *et al*., 2008; Herben *et al*., 2017; Van Drunen & Husband, 2019), each of which suggests that MCE impacts the ability of polyploids to establish. Given that rates of uniparental reproduction will vary among species and populations (Whitehead *et al*., 2018), the strength of MCE will also vary, leading to different probabilities of establishment depending on the reproductive traits that a new polyploid possesses and the ecological context in which it arises.

While theoretical benefits of uniparental reproduction for polyploid establishment are clear and the empirical support is broad, studies of the association of specific reproductive traits with polyploidy, such as the capacity for self‐pollination, have sometimes shown relatively weak or even contradictory associations (Mable, 2004; Husband *et al*., 2008; Glick *et al*., 2016). The lack of a strong association between polyploidy and reproductive traits has led some to question the importance of MCE for polyploid establishment (Mable, 2004; Van Drunen & Husband, 2019; Van De Peer *et al*., 2021). We argue that the ongoing debate is fueled in part by the lack of a comprehensive framework describing the expected relative contribution of self‐reproductive traits for reducing MCE. Because different uniparental reproductive strategies can impact MCE in similar ways, placing these in a common framework can help inform interpretations of findings, suggest overlooked research directions, and help develop clear hypotheses for how polyploidy and uniparental reproductive traits are associated.

The framework and theory around the role of MCE in preventing polyploid establishment has mainly been developed in the context of the establishment of autopolyploids (i.e. polyploids formed within a species, Box 1, Levin, 1975; Rausch & Morgan, 2005; Van Drunen & Friedman, 2022, although see Fowler & Levin, 2016), despite the fact that allopolyploids (i.e. polyploids formed by hybridization between species) are likely at least as common as autopolyploids in nature (Barker *et al*., 2016). Because allopolyploids more often are described as a distinct species than autopolyploids (Barker *et al*., 2016), allopolyploids probably weigh heavily in analyses of traits that are hypothesized to facilitate polyploid establishment and in analyses of polyploid biogeography (e.g. as in Rice *et al*., 2019). One underlying reason for the exclusion of allopolyploids from the MCE framework over the past 50 yr may be partly due to a lack of data on allopolyploid triploid fitness (Ramsey & Schemske, 1998); however, accumulated evidence suggests that triploid allopolyploids also have reduced fitness relative to diploids and tetraploids (e.g. through reduced fertility; Shepherd, 1999; Greiner & Oberprieler, 2012; Vallejo‐Marín *et al*., 2016). Thus, MCE should generally impact the establishment of both allo‐ and autotetraploids.

Understanding how plant reproductive traits and MCE impact polyploid establishment is broadly important to the field of polyploidy research because it informs predictions about whether and to what degree polyploid establishment requires fitness advantages or niche divergence (Clo *et al*., 2022). If the required fitness advantage needed for polyploids to establish varies across plant communities because of variation in MCE, polyploid frequencies could differ across environments and regions without the need to acquire adaptive traits, similarly to how communities of species can sort nonrandomly without adaptation (Hubbell, 2005). As angiosperm reproductive strategies often display strong spatial patterning, such as varying degrees of self‐pollination (Moeller *et al*., 2017; Rodger *et al*., 2021), understanding the role of uniparental reproduction in polyploid establishment will in turn enhance our understanding of the biogeographical patterns in polyploid frequency.

### Purpose and aims

The purpose of this Viewpoint article is to provide a broad framework for understanding the connections among reproductive traits associated with polyploid establishment. We start by discussing the mechanistic pathway of reduced fitness in triploids that causes MCE, and show how the expected association between polyploidy and uniparental reproduction will depend on triploid fitness and unreduced gamete (UG) production (see the Triploid fitness and unreduced gamete production determine the potential role of MCE section). We then discuss the relationship between polyploidy and uniparental reproduction (see The role of clonality and sexual reproduction in polyploid establishment section), highlighting how uniparental reproduction (i.e. clonality and self‐pollination) may show only weak correlation with polyploidy despite having a potentially important influence on polyploid establishment. In the Sexual reproduction: The crucial but overlooked importance of outcrossing to MCE section, we explore pathways for polyploid establishment through sexual reproduction. We emphasize the key role of pollinators in preventing polyploid establishment, and show in an agent‐based model how pollinators, together with self‐pollination traits, can interact to impact MCE (see the Animal pollinators as mediators of MCE section). We additionally review the impact of assortative mating and reproductive isolation on polyploid establishment (see The effect of assortative mating and reproductive isolation on MCE section), highlighting that little is known about how these processes influence minority cytotype populations. Finally, we discuss why self‐pollination traits (see The influence of self‐pollination and other plant traits on MCE section) may be important but often go undetected in studies on polyploid establishment. In the Predicted effects of variation in MCE on biogeographic patterns of polyploidy section, we discuss how variation in MCE could influence biogeographical patterns of polyploid frequency, and propose a framework to explore this connection. We provide three predictions that can be used to test two nonmutually exclusive hypotheses explaining biogeographical patterns in polyploids: (1) that variation in polyploid frequency is driven by differences in the strength of MCE among species and environments; or (2) that biogeographic patterns in polyploid frequency reflect adaptation to stressful environments. Overall, we hope this discussion contributes to the demystification of what is often considered the paradox of polyploid establishment and inspires new efforts to explore ecological and evolutionary processes favoring polyploids.

## Triploid fitness and unreduced gamete production determine the potential role of MCE

Diploids and tetraploids have strong postmating reproductive isolation because the fusion of haploid and diploid gametes yields few viable seeds, a phenomenon known as ‘triploid block’ (Burton & Husband, 2000; Köhler *et al*., 2010). In addition, even when triploids are viable, they are often of low fertility (Sax, 1922; Ramsey & Schemske, 1998). The degree of triploid block and triploid fertility vary among taxa (Ramsey & Schemske, 1998; Salony *et al*., 2024), and their combined effects set the stage for frequency‐dependent MCE (Levin, 1975). Because of their reduced fitness, triploids are generally rare in natural populations (Ramsey & Schemske, 1998). In addition, the rate of unreduced gamete (UG) production (e.g. the rate at which a diploid cytotype produces diploid pollen or ovules) is known to be critical to polyploid formation, but it also influences the strength of MCE. Estimates of rates of UG production are *c*. 1% for most species (median 1.2%: Kreiner *et al*., 2017), but UG production varies strongly among populations, among individuals and between sex functions, due to genetic and environmental effects (e.g. Mason *et al*., 2011; Kreiner *et al*., 2017).

**Fig. 1:**
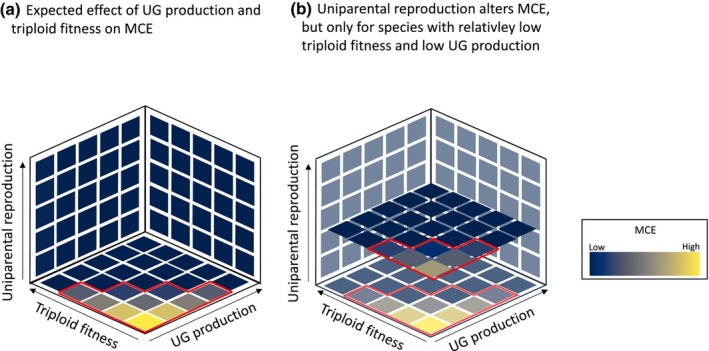
A conceptual illustration demonstrating that the frequency of uniparental reproduction will impact the strength of minority cytotype exclusion (MCE) only when triploid fitness and unreduced gamete (UG) production are low. (a) When triploid fitness, UG production and uniparental reproduction are low, MCE will be high and prevent polyploid establishment. By contrast, under high‐triploid fitness or UG production, MCE will be low regardless of the rate of uniparental reproduction (i.e. the back walls of the cube). (b) When triploid fitness and UG production are low, an increase in uniparental reproduction will reduce MCE. Red markings indicate the space influenced by MCE. The proportionately lower MCE when both triploid fitness and UG production are high is based on the documented production of high frequencies of UG by triploids. In this illustration, triploid fitness varies between 0 and 1, where 1 represents fitness equal to diploids.

Together, high UG production or triploid fitness can diminish MCE. Thus, it is only within the space of a low UG production and a low‐triploid fitness that other processes such as uniparental reproduction will play a role in influencing polyploid establishment (Fig. 1). Triploid fitness and UG production may in addition interact to disproportionately favor polyploid establishment through a high production of 2n and 3n gametes in triploids (more than twice as abundant as reduced haploid gametes; Ramsey & Schemske, 1998; Fig. 1).

## The role of clonality and sexual reproduction in polyploid establishment

Uniparental reproduction can be divided into two broad categories: clonal reproduction by vegetative growth or apomixis, and sexual reproduction via self‐pollination (Barrett, 2015). Regardless of which uniparental reproductive strategy a plant uses, MCE is expected to be weaker when uniparental reproduction is efficient, because this decreases reliance on outcrossing and thus reduces the probability of inter‐cytotype hybridization (Herben *et al*., 2017). Thus, in order to overcome MCE, clonal reproduction and self‐pollination can be viewed as complementary ways of supporting polyploid establishment.

**Fig. 2:**
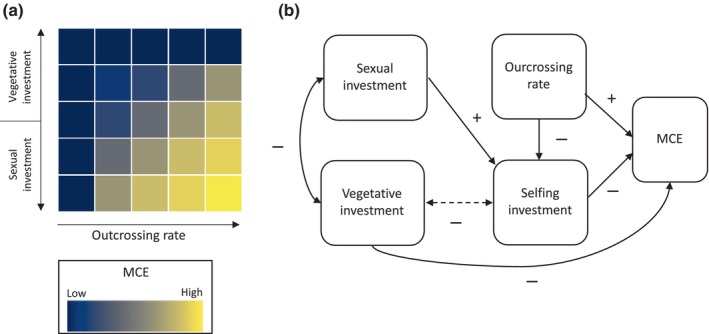
(a) A theoretical scheme showing how the two major forms of plant reproduction, vegetative or sexual, can impact the strength of minority cytotype exclusion (MCE). When plants mainly reproduce sexually, the strength of MCE will vary depending on the rate of outcrossing vs selfing. By contrast, when plants reproduce mainly vegetatively, the mating strategy will have little influence on MCE. (b) Whether plants mainly invest in vegetative or sexual reproduction is determined by fitness trade‐offs and these strategies will therefore covary negatively. The negative effect of vegetative investment on sexual reproduction will also negatively influence the investment in selfing (and vice versa). Thus, the effect of either vegetative reproduction or selfing is expected to mask the strength of the association of the other trait to polyploidy. Finally, because both vegetative reproduction and selfing provide reproductive assurance, these traits are sometimes also directly negatively associated (indicated by the dashed line), which can further obscure associations between these traits and polyploidy.

However, while both self‐pollination and clonal reproduction are common in polyploids (Barringer, 2007; Herben *et al*., 2017; Van Drunen & Husband, 2019) their respective contributions to weakening MCE can be obscured by their association (Fig. 2a). The allocation of resources to vegetative or sexual reproduction is determined by fitness trade‐offs (see reviews by Barrett, 2015; Yang & Kim, 2016). As a result of these trade‐offs, the contribution of vegetative reproduction and selfing to polyploid establishment can be expected to covary negatively (Van Drunen and Dorken 2012; Barrett, 2015). For example, Arctic plants generally show high frequencies of autonomous self‐pollination (Molau, 1992; Rodger *et al*., 2021), but those that reproduce clonally by bulbils often produce no seeds (e.g. Molau, 1992). Furthermore, both clonal reproduction and self‐pollination can provide reproductive assurance when outcrossing is unlikely. In Solanaceae, Vallejo‐Marín & O'Brien (2007) found a negative association between vegetative reproduction and self‐compatibility. The low occurrence of self‐compatibility among clonal species in Solanaceae indicates that there is not only a resource trade‐off, but also hints at a trait trade‐off between selfing investment and clonal reproduction (Fig. 2b, dashed line). This illustrates the possibility that high investment in vegetative reproduction will reduce the importance of selfing in facilitating polyploid establishment and vice versa. The negative correlation between vegetative reproduction and self‐pollination indicates a suppressed effect between the traits, where the more common form of uniparental reproduction will mask the predicted relative importance of the other (Laughlin & Grace, 2019; Fig. 2b). However, when a species is both clonal and self‐compatible, these traits can interact to facilitate polyploid establishment through geitonogamy (i.e. selfing between flowers on the same individual, see The effect of assortative mating and reproductive isolation on MCE section).

Species with widespread asexual reproduction often have a relatively high frequency of triploids (e.g. Karpavičienė, 2017; Chung *et al*., 2015), indicating that the triploid disadvantages mainly affect sexual reproduction rather than vegetative growth (Chrtek *et al*., 2017). In clonal species, the sterility and low‐seed viability of triploids have less impact on fitness because of a lower reliance on sexual reproduction (Barrett, 2015). Thus, MCE is generally expected to be weaker in species which reproduce clonally, which could help explain why the association between polyploidy and clonality often seem stronger than between polyploidy and self‐pollination (e.g. Mable, 2004; Van Drunen & Husband, 2019).

## Sexual reproduction: the crucial but overlooked importance of outcrossing to MCE

Although the ability to self has long been recognized as a means of facilitating polyploid establishment (Levin, 1975; Barringer, 2007), the influence of pollen vectors on reducing rates of establishment has received less attention. In the absence of biotic or abiotic pollen vectors, only clonal reproduction or autonomous self‐pollination are possible, thus avoiding the frequency‐dependent disadvantage of MCE. To explore the role of pollen vectors in mediating MCE, we focus on animal pollination, which is the dominant mode of pollination throughout the evolutionary history of angiosperms (Stephens *et al*., 2023).

Previous research has focused on the role of pollinators in facilitating polyploid establishment via assortative mating (Kennedy *et al*., 2006; Sutherland *et al*., 2020; see The effect of assortative mating and reproductive isolation on MCE section). However, it is important to remember that, together with the negative effects of triploidization, pollinators actually cause MCE. Any animal pollinator contributing to outcrossing is expected to increase MCE, as all other forms of reproduction are uniparental and therefore weaken MCE. At the same time, flower traits such as the stage of autonomous self‐pollination and the number of flowers will also influence how effective pollinators are at depositing outcross pollen compared to self‐pollen (Lloyd & Schoen, 1992), thus influencing the extent of MCE as a barrier to polyploid establishment. Below, we outline general expectations about the strength of MCE within populations and species based on plant–pollinator ecology. First, we detail how pollinators can influence the strength of MCE (see the Animal pollinators as mediators of MCE section). We then discuss the potential of assortative mating and reproductive isolation through pollinators to reduce MCE (see The effect of assortative mating and reproductive isolation on MCE section). Finally, we discuss how self‐pollination traits and floral traits influence MCE (see The influence of self‐pollination and other plant traits on MCE section). We discuss the expected outcomes of MCE on polyploids broadly, including both allo‐ and autopolyploids, but also acknowledge that MCE could act differently in these two forms of polyploids.

### Animal pollinators as mediators of MCE

Variation in the composition, abundance and behavior of pollinators impacts the deposition rates of self‐pollen, outcross pollen, and heterospecific pollen among plant species (Ne'Eman *et al*., 2010; Koski *et al*., 2018; Ashman *et al*., 2020), influencing rates of self‐pollination, and the overall success of seed production (Ashman *et al*., 2004; Fowler & Levin, 2016). As both the rate of self‐pollination relative to outcrossing and the proportion of sexual reproduction relative to vegetative reproduction impact MCE, pollinators' variable contribution to reproduction is expected to drive variation in polyploid establishment (Husband, 2000; Sutherland *et al*., 2020). High‐outcross pollen deposition can reduce the chances for polyploid establishment even in lineages with strong autonomous selfing ability through preceding self‐pollen deposition (e.g. Goodwillie & Weber, 2018; Whitehead *et al*., 2018; Xiao *et al*., 2021). Across angiosperms, realized selfing rates are about half of autonomous selfing rates (defined as the autonomous selfing capacity in the absence of pollinators) (Igic & Busch, 2013; Rodger *et al*., 2021), highlighting pollinators' potential role in shaping the ratio of outcrossed to selfed seeds (Goodwillie & Weber, 2018; Whitehead *et al*., 2018).

**Fig. 3:**
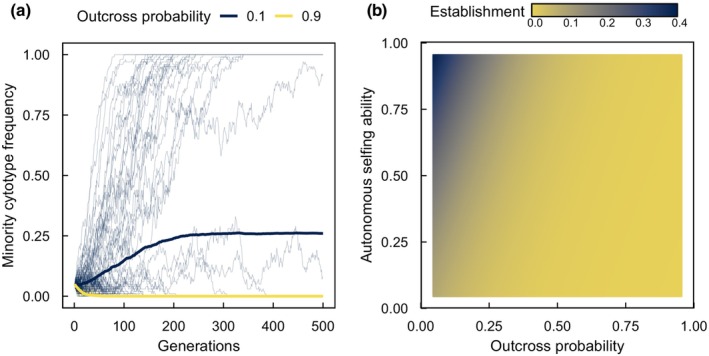
An agent‐based simulation model of a self‐compatible polyploid minority cytotype that examines how autonomous selfing interacts with pollinator‐mediated outcrossing to influence polyploid establishment. When outcross pollen deposition occurs before autonomous selfing it reduces polyploid establishment regardless of the autonomous selfing ability of polyploids. (a) A sample panel showing the proportion of polyploids (i.e. the minority cytotype) through time in individual simulation runs (thin lines), showing the outcome of 100 simulations, each with polyploids initially making up 5% of the population. Outcrossing rates were set to 0.1 or 0.9, and the autonomous selfing ability was set to 80%. Averages for each outcrossing rate are shown as thick lines. Fitness differences between the cytotypes are drawn from a normal distribution (mean = 0, SD = 2.5). (b) Summary of simulation outcomes over the full parameter space of autonomous selfing capacity and the probability of outcrossing. Triploid fitness and unreduced gamete production were set to 0 to focus on the interaction between pollen vectors influencing outcrossing rates and rates of self‐pollination. The results show that rates of outcrossing have a large influence on minority cytotype exclusion even when the capacity for autonomous selfing is high, because outcross pollen deposition reduces rates of self‐pollination. See Supporting Information Methods S1 for model details.

The key role of pollinators in determining the strength of MCE suggests that characteristics of the pollinator community will have an outsized effect on the probability of polyploid establishment. We developed an agent‐based simulation model to explore how autonomous selfing and the pollinator environment (e.g. the effectiveness of the pollinator community) interact to influence the strength of MCE, thereby influencing the rate of polyploid establishment (Fig. 3). In our model, diploids and polyploids have equal mean fitness, but polyploid fitness varies relative to diploid fitness (as a polyploidization effect; Bomblies, 2020). The model shows that the probability of minority cytotype establishment is not only influenced by the autonomous selfing ability, but also by the probability of outcross pollination. An additional insight from the model is that there is no need for polyploidization events to consistently lead to a net fitness increase to ensure establishment.

Pollinators possess a wide range of behaviors and functional traits associated with successful pollination and outcrossing. For example, pollinator specialization can increase intraspecific pollen transfer (Wei *et al*., 2021), and functional traits such as the pollinator's body size can influence the pollinators' efficiency of depositing outcross pollen (Ne'Eman *et al*., 2010; Koski *et al*., 2018). High flower visitation frequencies by specific bee species can increase pollen transfer between flowers (e.g. Koski *et al*., 2018). In the context of MCE, these properties of the pollinators as well as their abundances will impact how likely minority cytotypes are to receive pollen from the majority cytotype, thus impacting the probability of their establishment. Pollinator abundance and composition vary across plant communities and habitats, for example, due to environmental filtering (Elberling & Olesen, 1999; Orr *et al*., 2021), which can lead to reproductive outputs that differ among plant communities (Koski *et al*., 2018; Urbanowicz *et al*., 2018). As a consequence, the probability of polyploid establishment may be specific to the location a minority cytotype is found in.

The species richness of the pollinator community may also influence the strength of MCE, as a more species‐rich pollinator community can provide stability of pollination services across space and time, and buffer the effects of local extinctions of pollinator species (Waser *et al*., 1996). For example, pollen deposition may be reduced in plant populations where effective pollinators are missing, such as in isolated populations (e.g. on islands) or at the plant species' range limit (Moeller *et al*., 2012). If wind pollination is partly driven by the loss of pollinators (Friedman & Barrett, 2008; Soza *et al*., 2013) the high prevalence of polyploids in species transitioning from animal to wind pollination in the genus *Thalictrum* may reflect lower MCE during these transitions. Over evolutionary time frames, both local and regional pollinator extinctions could reduce MCE in plant communities, potentially promoting polyploid establishment. This is consistent with the observation of higher rates of polyploid establishment during and after mass extinction events (Freeling, 2017), a pattern previously attributed to polyploids' high tolerance to stressful environments (Vanneste *et al*., 2014, Van De Peer *et al*., 2021, though see Freeling, 2017).

Despite the potentially large impact of the pollinator community on MCE and the evolution of polyploidy in angiosperms, few studies have investigated these relationships. The two studies that have explicitly investigated the role of pollinators in mediating MCE found that pollinators played a crucial role (Husband, 2000; Sutherland *et al*., 2020). Several studies have explored the role of pollinators in *facilitating* polyploid establishment through assortative mating, but overlooking the role of pollinators specifically in *preventing* polyploid establishment leads to an incomplete understanding of the factors influencing polyploid ecology and evolution, in particular when considering how changes in the pollinator community can impact polyploid establishment.

### The effect of assortative mating and reproductive isolation on MCE

High costs of inter‐cytotype matings experienced disproportionately by the rare cytotype causes MCE. Assortative mating, in which within‐cytotype crosses occur more often than expected by chance, has the potential to reduce or eliminate the expected negative impact of inter‐cytotype mating, thus reducing MCE and increasing the probability of polyploid establishment (Gross & Schiestl, 2015; Sutherland *et al*., 2020). Assortative mating can take place for a number of reasons, for example due to closer spatial proximity or greater overlap of flowering phenology in individuals of the same cytotype compared to individuals of different cytotypes (Baack, 2005; Barrett, 2015), or due to pollinator preferences or pollinator constancy for different cytotypes (Kennedy *et al*., 2006; Mortier *et al*., 2024). Models have shown that these types of assortative mating can strongly favor polyploid establishment (Oswald & Nuismer, 2011; Van Drunen & Friedman, 2022; although see Clo *et al*., 2022). For example, pollinators may prefer the typically larger flower sizes in polyploids, thus increasing the likelihood of these polyploids being visited (Gross & Schiestl, 2015; more on assortative mating and plant traits in The influence of self‐pollination and other plant traits on MCE section). Niche differentiation, seed dispersal patterns and mode of vegetative reproduction can also impact assortative mating. For example, reproduction through clonal ramets influences the proximity of same‐cytotype individuals, and thus increases the probability of assortative mating through geitonogamy (Baack, 2005; Vallejo‐Marín *et al*., 2010; Baniaga *et al*., 2020; Mortier *et al*., 2024). Clonal growth thus facilitates polyploid establishment both through the direct avoidance of sexual reproduction and through increased self‐pollination (if self‐compatible) and assortative mating, highlighting the potential for interactions among processes that impact MCE. The opportunity for assortative mating may also be higher in allopolyploids, for example through the attraction of novel pollinators through changes in floral traits from hybridization (Peng *et al*., 2024).

The contribution of pollinator preferences to assortative mating has been widely studied, though mainly in autopolyploids, with mixed results: pollinators sometimes have cytotype‐specific preferences (Kennedy *et al*., 2006; Gross & Schiestl, 2015) while at other times not (Castro *et al*., 2020; Schmickl *et al*., 2024). These studies highlight how variation in the behavior of pollinators can potentially facilitate polyploid establishment for plants; however, pollinator‐induced assortative mating alone is unlikely to achieve polyploid establishment. For example, Sutherland *et al*. (2020) revealed that, while minority cytotypes benefited from assortative mating and increased visitation rates, the minority cytotype population (1 : 3 frequency to the majority cytotype) still suffered a strong reduction in seed set. This is because even a strong pollinator preference for a minority cytotype still will lead to higher absolute deposition of the majority cytoype's pollen (Fowler & Levin, 2016). Studies using small frequency differences between diploids and polyploids (Kennedy *et al*., 2006; Gross & Schiestl, 2015) may therefore exaggerate the effects of assortative mating to polyploid establishment. Thus, it is important to consider the influence of assortative mating on small populations specifically. Moreover, given that most pollinators visit a wide range of plant species, we generally should not expect large differences in visitation between cytotypes (Waser *et al*., 1996). Thus, assortative mating probably has to stem from multiple sources and not pollinator preferences alone (e.g. phenological differences, pollinator preferences; Castro *et al*., 2020) to overcome MCE.

Prezygotic isolation through assortative mating and postzygotic isolation give an estimate of total reproductive isolation (RI) between cytotypes (Castro *et al*., 2020; Sutherland *et al*., 2020). While RI is argued to favor polyploid establishment, prezygotic and postzygotic RI have fundamentally different effects on MCE. There are two reasons why postzygotic RI does not necessarily favor polyploid establishment. First, as previously discussed, cytotype hybridization can facilitate polyploid establishment rather than reducing it (i.e. through a triploid bridge). Second, the production of same‐cytotype offspring can still be strongly reduced in minority populations due to MCE even under complete postzygotic RI, as found by Sutherland *et al*. (2020), because of insufficient pollen loads from the same‐cytotype (Fowler & Levin, 2016). In other words, a complete postzygotic reproductive barrier between cytotypes can still lead to strong MCE and hinder polyploid establishment if all the received pollen comes from a different cytotype. To develop a better understanding of the role of assortative mating and RI in polyploid establishment, it is therefore necessary to consider both how postzygotic RI can reduce the potential of a cytotype to overcome MCE, as well as how prezygotic RI can increase it.

### The influence of self‐pollination and other plant traits on MCE

**Fig. 4:**
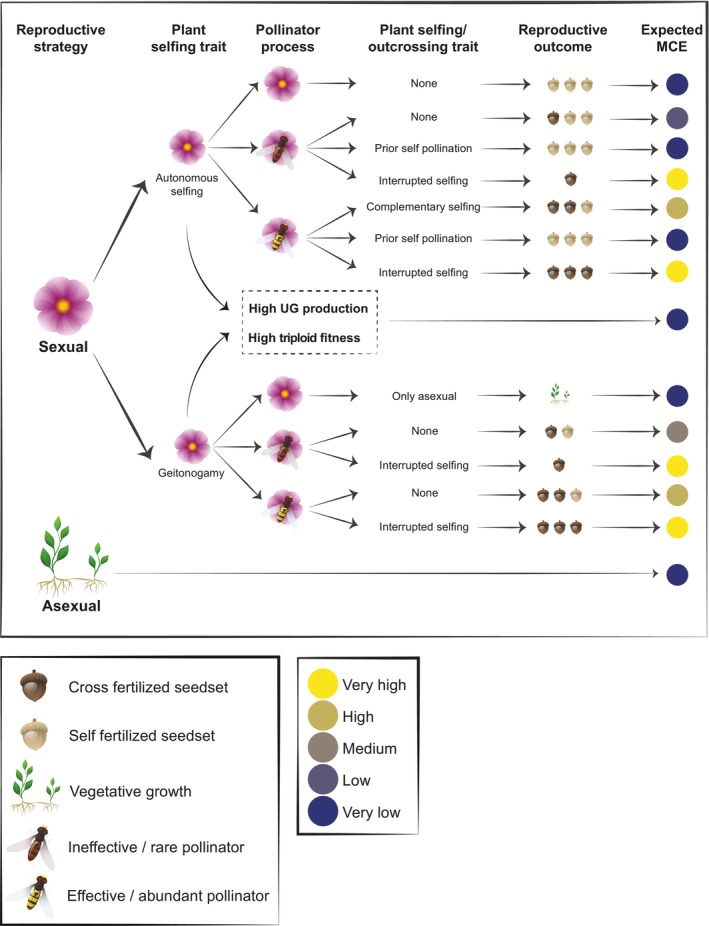
A framework illustrating the contribution of vegetative reproduction, triploid fitness, unreduced gamete (UG) production, as well as the efficiency of pollinators and self‐pollination rates to polyploid establishment. The importance of sexual strategy will interact with pollinators and specific plant traits to determine the strength of minority cytotype exclusion (MCE). Autogamous self‐pollination may lead to polyploid establishment more often than geitonogamous self‐pollination; however, both modes will vary in their potential to establish polyploids depending on pollinators. Additionally, the timing of self‐pollination (before, complementary to, or interrupted by outcross pollen; Lloyd & Schoen, 1992) will impact MCE by affecting the number of ovules available for self‐pollination (e.g. Goodwillie & Weber, 2018; Xiao *et al*., 2021). In this framework, effective and ineffective pollinators were assumed to be equal in their deposition of self‐pollen but differ in their deposition of outcross pollen.

Self‐pollination has the potential to facilitate polyploid establishment in sexually reproducing species by reducing MCE through inter‐cytotype mating. Polyploidization can also weaken gametophytic self‐incompatibility systems acting through self‐pollen recognition, thereby increasing the frequency of self‐pollination among polyploids (Stone, 2002). Allopolyploids can be particularly susceptible to becoming self‐compatible, as the introduction of a broken self‐incompatibility system from one ancestral species can be sufficient to promote self‐compatibility (Kolesnikova *et al*., 2023). Furthermore, polyploidy can weaken inbreeding depression caused by selfing, which may allow new polyploids to be stronger competitors than their diploid progenitors (Clo & Kolář, 2022; Orsucci *et al*., 2022). Indeed, several recent case studies reveal transitions from self‐incompatibility in diploid progenitors to self‐pollination in polyploids (Orsucci *et al*., 2022; Kolesnikova *et al*., 2023; Novikova *et al*., 2023). However, despite the expected advantages of self‐pollination to polyploid establishment, broad associations between polyploidy and selfing are often relatively weak (Mable, 2004; Barringer, 2007), particularly in autopolyploids (Husband *et al*., 2008). Small differences in self‐incompatibility among diploids and polyploids as well as the positive association between polyploidy and dioecy (Glick *et al*., 2016, but see Osterman *et al*., 2024) seemingly contradict the idea that self‐pollination is important to polyploid establishment. The weak relationship between self‐pollination and polyploidy may partly be explained by factors discussed previously (see The role of clonality and sexual reproduction in polyploid establishment section). In addition, we argue that even if self‐pollination is important to overcome MCE, the theoretical underpinnings of MCE do not always suggest a strong relationship between polyploidy and self‐pollination and associated traits (e.g. in line with findings of Mable, 2004; Barringer, 2007; Fig. 4).

While we can expect that polyploids should rely on self‐pollination, predictions for the frequency of selfing in diploids and polyploids within lineages may be quite similar. For example, polyploid formation may be more successful in lineages where diploids are selfing if this means newly formed polyploid individuals can also self‐pollinate. Because both cytotypes self‐pollinate, the correlation between polyploidy and self‐pollination will be weak. Furthermore, because polyploids will be more successful in their establishment when they originate from diploid selfers, assessments of the self‐pollination–polyploidy relationship that correct for the phylogenetic relatedness among species will underestimate the role of self‐pollination in polyploid establishment. Instead, the phylogenetic correction will interpret high rates of polyploids in selfing lineages as a consequence of relatedness (Westoby *et al*., 2023), and effectively give increased weighting to polyploids that overcome MCE in other ways, for example through a reduced triploid block, high UG production, or through pathways allowing plants to bypass self‐incompatibility (e.g. the mentor effect, Desrochers & Rieseberg (1998) where bypassing self‐incompatibility is facilitated by hybrid pollen). We should thus not expect the relationship between polyploidy and self‐pollination to be strong even when self‐pollination plays an important role in determining the success of polyploid establishment.

The complex relationship between self‐pollination and polyploidy highlights the need for a comprehensive predictive framework of MCE to develop accurate models and predictions of polyploid establishment. Without such a framework, noncausal correlations between polyploidy and other traits may also be misinterpreted due to functional correlation. For example, although studies find a positive relationship between perennialism and polyploidy (e.g. Rice *et al*., 2019; Van Drunen & Husband, 2019), perennialism is highly correlated with clonal reproduction. Among nonclonal species, annuals and perennials have similar frequency of polyploidy (Van Drunen & Husband, 2019). The proposed mechanism linking perennialism and polyploidy is that perennialism increases the temporal overlap of rarely formed polyploids, enhancing mating opportunities (Van Drunen & Husband, 2019). However, perennial plants recruit new individuals more slowly, resulting in fewer offspring per year compared to annuals. The probability of producing offspring will be proportionately smaller for a perennial plant because of slower turnover of individuals in a population. In addition, recent models suggesting that perennialism favors polyploidy rely on the assumption that perennialism increases the total fitness of polyploids through additional reproductive seasons (Van Drunen & Friedman, 2022). Thus, the hypothesis that polyploidy is favored by perennialism should be interpreted with caution. In Fig. 4, we present a framework in which several traits of plants and pollinators interact to influence the expected strength of MCE in plant populations. In this example, we explore how the type and timing of self‐pollination interacts with pollinator presence/efficiency to influence the expected strength of MCE. This framework can be expanded to include additional traits related to self‐pollination or outcrossing, such as flower count and proximity of stamens to a stigma (herkogamy).

Floral traits such as display size and nectar rewards are essential for plants to attract pollinators to achieve cross pollination. While some traits such as short styles, few flowers and small petals are broadly associated with self‐pollination (Lanuza *et al*., 2023), they may have weak associations with polyploidy for two reasons. First, while selfing is associated with reduced petal size, polyploidization events can increase petal size (i.e. due to the larger cell size of polyploids; Porturas *et al*., 2019). Therefore, despite the broad association of self‐pollination with small flowers, polyploidization can confound any association in this direction. In addition, the larger reproductive structures such as larger stamens and stigmas, could allow for differential pollen placements on the pollinator's body and for more effective pollen deposition (Minnaar *et al*., 2019; García‐Muñoz *et al*., 2023), thus providing alternative pathways to overcome MCE through assortative mating. Second, the associations between self‐pollination and floral traits are generally weak across angiosperms because the selection for traits is context dependent. For example, based on the evidence that autonomous selfers have reduced flower numbers, we might predict more frequent polyploid establishment in lineages with low flower numbers (Lanuza *et al*., 2023). However, such predictions are likely to yield weak relationships because geitonogamous species rely on high floral attraction and higher flower numbers for self‐pollination. Such context dependencies may explain why it is difficult to find specific polyploidy–selfing trait associations (Jiménez‐Lobato *et al*., 2019).

In summary, even if self‐pollination is predicted to have a large impact on polyploid establishment (Husband, 2000; Sutherland *et al*., 2020), the detection of strong associations between polyploidy and selfing capacity or selfing‐related traits is unlikely. However, instead of indicating that a specific trait is not associated with polyploid establishment, the weak associations of self‐pollination traits with polyploidy may reflect the complex relationships among sets of traits that impact the success of polyploids. For example, while the positive polyploid–dioecy association seems to go against theoretical expectations, evidence that this association arises from polyploidization favoring dioecy (and not the reverse) both resolves the apparent contradiction (Osterman *et al*., 2024) and suggests that subsequent evolution may obscure how MCE impacts polyploid establishment. Thus, inferring the impact of MCE on polyploid establishment can depend on the causal direction between polyploidy and a trait of interest (i.e. which trait causes which), highlighting the importance of predicting how trait combinations relate to polyploidy. The role of self‐pollination may similarly become more apparent when assessed in combination with properties of the pollinator community, the importance of vegetative reproduction, and the strength of triploid block (Fig. 4).

## Predicted effects of variation in MCE on biogeographic patterns of polyploidy

Variation in the frequency of polyploids across the globe has been attributed to polyploids having higher tolerance to abiotic stress (Van De Peer *et al*., 2021). While polyploids are sometimes found to be stress‐tolerant (Bomblies, 2020), the evidence that abiotic stress tolerance broadly drives polyploid distribution is relatively weak. Studies on eco‐evolutionary niche shifts suggest diverse but lineage‐specific impacts of polyploidy (Padilla‐García *et al*., 2023). Thus, a framework incorporating additional mechanisms may shed light on this biogeographic pattern. For example, both Gustafsson (1948) and Molau (1993) observed that Arctic polyploids were more dependent on uniparental reproduction than diploids and hypothesized that this dependency caused the high polyploid frequencies in the region. Molau also observed that Arctic polyploids are confined to more stable late‐thawing habitats (Molau, 1993) that are buffered from extreme temperatures (Björk & Molau, 2007), in seeming contradiction to the polyploidy–stress association hypothesis.

If uniparental reproduction and pollination regimes differ systematically between regions and habitats, we can expect the strength of MCE to also vary across the globe. Specifically, regions with relatively high uniparental reproduction would be predicted to have weaker MCE and higher frequencies of polyploids. In fact, pollinator communities are known to vary among geographic regions, which could impact the rate of self‐pollination (Orr *et al*., 2021). For example, in the northern hemisphere, the relative richness of Dipteran pollinators increases as the richness of bees declines from mid to high latitudes (Elberling & Olesen, 1999; Orr *et al*., 2021). Furthermore, plants' adaptation to autonomous self‐pollination (i.e. autonomous selfing) and realized selfing rates increase at higher latitudes (Moeller *et al*., 2017; Rodger *et al*., 2021). In addition, the frequency of perennial herbs, a life‐history trait which is closely related to vegetative reproduction (Gustafsson, 1948; Van Drunen & Husband, 2019), also increases at high northern latitudes (Rice *et al*., 2019). At finer scales, different vegetation types within climatic regions may differ in the strength of MCE because they differ in the importance of specific modes of reproduction (Rice *et al*., 2019). For example, meadow communities often have high rates of vegetative reproduction compared to forest communities (Sammul *et al*., 2004), and thus these habitats likely differ in the strength of MCE and the likelihood of polyploid establishment across species.

Here, we propose a framework to evaluate the hypothesis that polyploid biogeography is driven by variation in MCE vs tolerance to abiotic stress (referred to as the ‘MCE‐biogeography hypothesis’, MBH, and ‘Stress‐biogeography hypothesis’, SBH, respectively). If variation in polyploid frequency is driven by the degree of tolerance to abiotic stress (SBH), we could expect to see variation in polyploid frequency even at small spatial scales depending on the stress experienced in the microsite. By contrast, if variation in MCE drives the distribution of polyploids (MBH), then gradients in uniparental reproduction and pollinator communities could be more important predictors of polyploid frequency. In many cases, expectations may be similar between SBH and MBH for polyploid distributions. For example, the high prevalence of polyploid species in recently deglaciated areas has been interpreted as evidence of polyploids' tolerance of stressful environments (Van De Peer *et al*., 2021), but the increased ability of polyploids to self‐pollinate and vegetatively reproduce (Razanajatovo *et al*., 2016) could cause a similar pattern. The fact that SBH and MBH may predict similar patterns for polyploid distribution highlights the need for tests in which their predictions differ. Below, we suggest three predictions for testing which hypothesis contributes more to biogeographical patterns of polyploidy. However, we also acknowledge that making falsifiable predictions to test whether SBH drives polyploid biogeography is challenging due to uncertainty over what constitutes ‘stressful’ for a plant; for example, cold or warm temperatures may induce stress (Bomblies, 2020). Likewise, we have not incorporated rates of UG production into our hypotheses, as UG production is also influenced by both warm and cold temperatures (Mason *et al*., 2011; Deng *et al*., 2024) and has not been found to be higher in Arctic plants (Eidesen *et al*., 2024).

### Prediction I

**Fig. 5:**
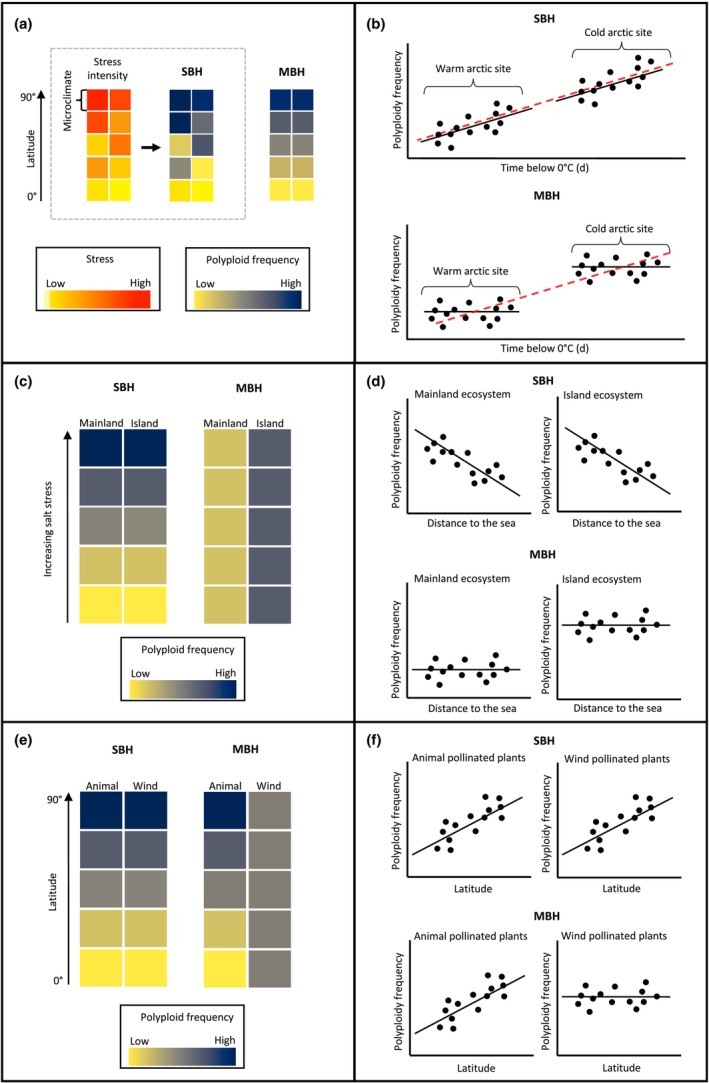
(a–f) A framework for evaluating the hypotheses that polyploid biogeography is driven by variation in minority cytotype exclusion (MCE) vs tolerance to abiotic stress (referred to as MBH (MCE‐biogeography hypothesis), and SBH (Stress‐biogeography hypothesis)). Spatial patterns of polyploid frequency in relation to various drivers are depicted in (a, c, e), while examples of the resulting predicted relationships between each driver and polyploid frequency are shown in (b, d, f). Each point in the linear relationship is a microsite or local community. The dashed red lines in (b) represent the relationships between sites, which is expected to be the same for SBH and MBH.

If the high frequency of polyploids at high latitudes is best explained by environmental stress, polyploids should be more successful (i.e. at higher frequencies or in greater abundance) at microsites that experience more stress (SBH, Fig. 5a,b). In the Arctic, plants experience wide temperature ranges of > 30°C over small spatial scales, and not all plants may experience freezing temperatures (Björk & Molau, 2007), making the Arctic an optimal system to study polyploidy–stress associations. By contrast, if MCE drives polyploid distributions (MBH, Fig. 5a,b), we expect no relationship between local minimum temperatures and polyploidy (Fig. 5b, black lines) because self‐pollination is not expected to correlate with the exposure to cold temperatures at the microsite scale. Note, however, that among regions, MCE may be weaker in colder Arctic communities because reduced pollinator services favor higher uniparental reproduction, leading to a higher polyploidy frequency at higher latitudes (as indicated between plots Fig. 5b, red line).

### Prediction II

Polyploids are often more resilient to salt stress than related diploids (Bomblies, 2020) and are also prevalent in island ecosystems (Vamosi *et al*., 2007; Rice *et al*., 2019; Meudt *et al*., 2021). According to the SBH, the establishment of polyploids on oceanic islands could be favored if a greater proportion of habitats is exposed to salt stress (e.g. due to a greater ratio of coastal to inland habitats). Alternatively, according to the MBH, the greater reliance of island colonizers on uniparental reproduction (Grossenbacher *et al*., 2017) may underlie the overrepresentation of polyploids on islands. Given that the coastal mainland is exposed to the same salt stress as on islands, comparing polyploidy frequencies on oceanic islands to mainland ecosystems could allow us to disentangle the degree to which polyploid distributions are driven by environmental stress vs reproductive processes. If environmental stress drives polyploid distributions, polyploid frequencies should increase with proximity to the sea in both island and mainland ecosystems (SBH, Fig. 5c,d). By contrast, if reproduction drives polyploid distributions, the distance to the coast will have little effect on polyploidy. Instead, the frequency of polyploids is expected to be higher on islands than in mainland ecosystems due to lower MCE, driven by impoverished pollinator communities and an establishment advantage from uniparental reproduction on islands (MBH, Fig. 5c,d).

### Prediction III

The conditions for sexual reproduction deteriorate with latitude more strongly in animal‐pollinated than wind‐pollinated plants (Rech *et al*., 2016; Rodger *et al*., 2021). Therefore, if MCE is an important determinant of polyploid establishment, the latitudinal gradient of polyploidy should be stronger in animal‐pollinated than wind‐pollinated plants, (MBH, Fig. 5e,f). While vegetative reproduction may be beneficial in adverse habitats regardless of pollination syndrome (Hautier *et al*., 2009), the reduction in species richness of pollinator communities would make animal pollination even more disadvantageous in Arctic regions (Elberling & Olesen, 1999; Orr *et al*., 2021). By contrast, if latitudinal patterns are driven by adaptation to extreme environments, we would expect the latitudinal change in polyploid frequency to be equal across pollination syndromes (SBH, Fig. 5e,f).

## Conclusion

Polyploids can overcome MCE through five pathways: high‐triploid fitness, UG production, clonal reproduction, self‐pollination and assortative pollination. Studies have provided evidence that each of these pathways affects polyploid establishment. However, to understand when and to what extent MCE acts as a barrier to establishment, the pathways must be considered together. Our agent‐based model reveals that pollinators can play a crucial role in polyploid establishment, as they are the main vector of outcross pollen, thus causing MCE. Therefore, communities of pollinators and their traits will also ultimately impact MCE, though this remains unexplored.

Because plants' reproductive strategies vary across the globe, the reliance of polyploids on uniparental reproduction to establish will likely influence their global distribution. Our proposed framework enables us to distinguish the effect of MCE from that of abiotic stress, suggesting that polyploids either should be more associated with environmental stress at a small scale, or with pollinator‐poor environments and uniparental reproduction. Future studies on understanding the success of polyploids among angiosperms should focus on the interactions between pollinator communities, uniparental reproduction, triploid viability, and UG production to understand how polyploids overcome MCE.

A greater understanding of polyploid establishment under different reproductive conditions will not only shed light on polyploid biogeography, but also give insight into how variation in reproductive strategies broadly influences the evolutionary trajectories of plants. While outcrossing is considered central to the success of angiosperms, the role of self‐pollination and vegetative reproduction in evolutionary patterns is underexplored and often seen as an obstacle (Igic & Busch, 2013). By contrast, their association with polyploidy suggests that self‐pollination and vegetative reproduction have played a more significant role in angiosperm evolution than is commonly assumed.

## Author contributions

WHAO conceived the ideas and wrote the first manuscript draft. JGH and WHAO conceptualized the agent‐based model, and JGH developed the agent‐based model. WHAO, JW and ADB wrote the manuscript. All authors contributed with conceptualization of the text and figures, and edited the manuscript.

## Disclaimer

The New Phytologist Foundation remains neutral with regard to jurisdictional claims in maps and in any institutional affiliations.

## Supporting information


**Methods S1** Agent‐based model structure.
Please note: Wiley is not responsible for the content or functionality of any Supporting Information supplied by the authors. Any queries (other than missing material) should be directed to the *New Phytologist* Central Office.

## Data Availability

No empirical data were used for this manuscript. The code for the agent‐based model is available at https://github.com/haganjam/Polyploidy_establishment.
