# Supplementary material for: The ecology of polyploid establishment and exclusion, with implications for polyploid biogeography
Source: New Phytol. 2025 Feb 10;246(1):47–60. doi: 10.1111/nph.20451 (PMC11883057; doi:10.1111/nph.20451)
Supplement: Supplementary file 1 — Methods S1 Agent‐based model structure. Please note: Wiley is not responsible for the content or functionality of any Supporting Information supplied by the authors. Any queries (other than missing material) should be directed to the New Phytologist Central Office. [file NPH-246-47-s001.pdf]

New Phytologist Supporting Information
Article title: The ecology of polyploid establishment and exclusion, with implications for polyploid biogeography
Authors: Wilhelm H. A. Osterman, James G. Hagan, Jeannette Whitton, Anne D. Bjorkman
Article acceptance date: 15 January 2025

Methods S1: Agent-based model structure

In the model, we initialize a set of 100 sites each occupied by an individual plant. The plants are randomly assigned one of two cytotypes: diploid or polyploid (i.e. a tetraploid for simplicity). All plants are fully self-compatible, with selfing assumed to be autonomous, and to take place after outcrossing. At the start of each simulation, polyploids are the minority cytotype and occupy 5% of the sites. At each time step, a proportion of individuals are randomly selected to be cross-pollinated according to the outcrossing rate parameter for that set of simulations. For each individual to be pollinated, a random individual from the remaining community is selected to pollinate it. Within-cytotype matings always produce individuals of the same cytotype (i.e., there are no unreduced gametes in our model). The number of seeds produced by intra-cytotype matings is controlled by a fitness parameter and a pollinator effectiveness parameter. Seed set is the product of the fitness parameter (i.e. potential seed set) multiplied by the pollinator effectiveness. For crosses between diploids, we set the fitness parameter to eight and the pollinator effectiveness to 80%. For crosses between polyploids, we set the fitness parameter to have the same mean as diploids, but with a standard deviation of 2.5. This means that, for a given simulation, the crosses between polyploids might produce more or fewer seeds but on average, across simulations, there is no difference in seed set from crosses between polyploids and between diploids. Intercytotype crosses were modelled as inviable.

For both types of intra-cytotype crosses, we control the proportion of seeds that can be set via autonomous selfing with an autonomous selfing ability parameter, which essentially determines the probability than an ovule that was not outcrossed will undergo autonomous selfing. Using this selfing ability parameter, we calculate the selfed seed set as the product of the fitness parameter and the selfing ability parameter. The selfed seed set is added to the outcrossed seed set, but the total seed set for a given individual cannot exceed the total specified as the fitness parameter. For individuals that were not selected for cross-pollination, all seeds come from selfing, and we follow the same procedure as above to calculate seed set from autonomous selfing.

Once all individuals in the population have set seed either through outcrossing, selfing or both, the seeds are assembled into a global seed pool. We then randomly select a proportion of plants to die (10% in our simulations), and replace these by random draws from the global seed pool. As a result, the model implicitly assumes zero-sum dynamics whereby every individual that

dies is replaced by a new individual, and the overall number of individuals remains constant (as per Hubbell 2001). Each simulation was run for 500 time-steps. We tracked the changes in the frequency of diploid and polyploid cytotypes and, by varying the parameters for outcrossing rate, fitness and selfing, study how these factors and their interactions affect MCE.

Using this model, we simulated 1000 runs each for 361 different combinations of outcrossing rate and the selfing ability parameter, both of which varied between 0.05 and 0.95. In **Fig. 3a**, an example set of simulations is shown. Specifically, this panel shows the results of 100 model runs (for clarity) with an outcrossing rate of 0.1 and 100 model runs with an outcrossing rate of 0.9. In both cases, the selfing ability parameter was set at 0.80 which means that 80% of an individual's non-outcrossed seeds will be set by autonomous selfing. We find that when the outcrossing rate is low and the selfing rate is high then, in many cases, polyploid cytotype can overcome MCE even without any fitness differences on average. However, with a high outcrossing rate, the polyploid cytotype did not overcome MCE in any of the 100 model runs. These results are generalized in **Fig. 3b.**, which summarizes simulation runs over the 361 parameter combinations that we explored, with the surface smoothed using a beta-regression model. Specifically, the model shows that, even when the selfing ability parameter is high, if outcrossing is too high (more than ca. 0.3 in this model), polyploids are unlikely to overcome MCE. Therefore, we show that polyploid establishment is strongly affected by how effectively pollen vectors achieve outcrossing. Because outcrossing processes can strongly impact selfing, and thus MCE, it is critical to include this perspective in studies attempting to explain polyploid establishment.
